# Aligning national human resource development with future workforce transitions and the SDGs: policy lessons from Thailand's research-funding system

**DOI:** 10.3389/frma.2026.1830772

**Published:** 2026-09-21

**Authors:** Pennee Narot, Narong Kiettikunwong, Siwaporn Fongthong, Pongmanut Deeod

**Affiliations:** 1 Faculty of Education, Khon Kaen University, Khon Kaen, Thailand; 2 College of Public Affairs and Policy, Khon Kaen University, Khon Kaen, Thailand; 3 Faculty of Economics, Khon Kaen University, Khon Kaen, Thailand; 4 Faculty of Humanities and Social Sciences, Khon Kaen University, Khon Kaen, Thailand

**Keywords:** innovation policy, national-level HRD strategy, policy-system learning, research-funding governance, SDG alignment, workforce transitions

## Abstract

**Background:**

National human resource development (HRD) strategies are increasingly linked to science, research, and innovation funding systems as mechanisms for building workforce capabilities and advancing Sustainable Development Goals (SDGs). Thailand's establishment of Program Management Units (PMUs) under the Science, Research and Innovation Plan (2020–2025) offers a significant empirical case for examining how mission-oriented funding intermediaries can steer capability formation in a middle-income economy.

**Objective:**

This study analyses how Thailand's PMU-based research-funding system has shaped national human-capital development in relation to future workforce transitions and SDG-oriented priorities, and derives transferable policy lessons for countries in comparable institutional contexts.

**Methods:**

An interpretive policy-analysis approach was employed, re-analyzing findings from a completed national monitoring study covering Programmes P1–P5 and P16. The original study used a mixed-methods design integrating quantitative performance assessment—including descriptive statistics, weighted mean scoring, and the Index of Item-Objective Congruence (IOC)—with qualitative methods comprising focus-group discussions, in-depth interviews structured around the McKinsey 7S framework, documentary analysis, and participatory review sessions.

**Results:**

Strong structural alignment was observed between the PMU programme objectives and national HRD priorities. The quantitative evaluation of Key Results across 376 funded projects demonstrated that 100% achieved its out-come alignment, with an overall performance score of 3.62, indicating a good level of performance. Mission-oriented funding logics, flexible intermediary coordination, and relational governance practices were identified as critical enabling factors. However, indicators requiring cross-agency integration demonstrated weaker performance. Persistent gaps include indicator fragmentation, over-reliance on output-based metrics, limited cross-programme coordination, and absence of longitudinal tracking mechanisms for sustained capability outcomes.

**Conclusions:**

Thailand's PMU-based model functions as a strategic governance instrument within the National Innovation System, co-producing capability formation through funding architecture, intermediary institutions, and relational governance. Effective alignment between research funding, workforce transitions, and SDGs requires three complementary elements: mission-oriented funding structures embedding workforce priorities within innovation policy; intermediary institutions capable of adaptive coordination; and dual-layer performance architectures combining output accountability with developmental evaluation. These lessons offer transferable relevance for middle-income and transition-economy contexts seeking to harness research investment for sustainable and inclusive human-capital development.

## 1 Introduction

Across many countries, the governance of science, research, and innovation funding has come to play an increasingly strategic role in shaping national human resource development (HRD) trajectories, particularly in the context of rapid technological change, labor-market transformation, and the global commitment to the Sustainable Development Goals (SDGs) (United Nations, 2015). Rather than viewing research funding merely as a mechanism for supporting academic knowledge production, contemporary policy frameworks increasingly position funding systems as part of a broader capability-formation architecture, linking investment decisions to skills development, workforce readiness, and the institutional conditions that enable economies to adapt to emerging technological and societal transitions (Edler and Fagerberg, 2017). This shift reflects a growing recognition that national competitiveness, social resilience, and inclusive development depend not only on technological outputs, but also on the capacity of human-capital systems to respond to digitalisation, demographic shifts, sectoral restructuring, and evolving forms of work (OECD, 2019a; Nedelkoska and Quintini, 2018).

Thailand provides a particularly relevant case within this global landscape. Recent reforms of the national science, research, and innovation system including the establishment of Program Management Units (PMUs) as mission-oriented funding intermediaries under the 2020–2025 national plan represent an effort to position research-funding governance as a lever for workforce-relevant capability development (National Higher Education, Science, Research and Innovation Policy Council, 2019). Within this architecture, Programmes P1–P5 on workforce and lifelong learning, together with Programme P16 on system reform, were explicitly designed to connect research investments with the expansion of researcher pipelines, the strengthening of lifelong-learning ecosystems, and the development of innovation-ready labor forces. The national monitoring study on key results and performance indicators under these programmes provides a rich empirical basis for examining how indicator-driven policy steering, implementation practices, and institutional coordination shape the alignment between research funding, human-capital outcomes, and long-term workforce-development priorities [Science, Research and Innovation Strategy and Policy Mission Group, Thailand Science Research and Innovation (TSRI), 2021].

Since the 2019 reorganization of Thailand's higher-education and science ministries, national research and innovation policy has been coordinated by the Ministry of Higher Education, Science, Research and Innovation (MHESI), with strategic direction set by the National Higher Education, Science, Research and Innovation Policy Council and its secretariat, the Office of the National Higher Education, Science, Research and Innovation Policy Council (NXPO) (National Higher Education, Science, Research and Innovation Policy Council, 2019). Research and innovation funding is pooled in the National Science, Research and Innovation Fund (NSRF) and disbursed through a small number of specialized, semi-autonomous intermediary agencies, the PMUs, each responsible for a distinct strategic domain for example, PMU-A for area-based and community development, PMU-B for human-resource and research-institution development (the funding agency for the present study), and PMU-C for national competitiveness enhancement (NXPO, 2024; PMU-B, 2024). This architecture replaced an earlier, more fragmented system of direct block-grant allocation to individual universities and research councils, and reflects a wider regional pattern among middle-income countries of shifting toward mission-oriented, agency-based research-funding governance (OECD, 2023). Thailand's higher-education system is large and heterogeneous, encompassing research-intensive public universities, regional Rajabhat and Rajamangala university networks, and specialized research institutes, but has historically been characterized by comparatively low gross domestic expenditure on research and development, a concentration of research capacity within a small number of flagship institutions, and persistent shortages of research and innovation talent in STEM-related occupations (UNESCO, 2021; Vandeweyer et al., 2020). It is against this backdrop of institutional fragmentation, uneven research capacity, and an urgent national need to reskill the workforce for digital, green, and demographic transitions that the PMU reform and Programmes P1–P5 and P16 in particular was introduced.

At the same time, Thailand's experience also reflects a set of tensions common to many middle-income and transition-economy contexts. These include difficulties in translating investment outputs into durable capability outcomes, fragmentation across programmes and implementing actors, and challenges in linking short-term performance indicators with broader workforce transitions and SDG-oriented development objectives. Against this backdrop, this article uses the Thai case as a policy-learning lens through which to explore how national research-funding governance can be more effectively aligned with future-workforce transformation and sustainable-development priorities (Doner and Schneider, 2016; Flanagan et al., 2011). By situating the empirical findings within global debates on labor-market change, innovation-system governance, and capability formation, the paper seeks to generate transferable insights for countries seeking to strengthen the strategic coherence between research investment, human-capital development, and long-term workforce resilience.

## 2 Theoretical positioning

Government research funding is increasingly used not only to stimulate scientific advancement but also to develop human capital, enhance workforce capabilities, and accelerate progress toward the SDGs (Organisation for Economic Co-operation Development, 2015; United Nations, 2015). Research funding policies therefore serve purposes beyond supporting scientific discovery by promoting skills development, fostering interdisciplinary collaboration, strengthening innovation capacity, and improving labor market outcomes (Organisation for Economic Co-operation Development, 2015). Consequently, understanding how research funding policies contribute to the development of researchers, graduates, and skilled professionals has become an important area of policy research.

In addition, research funding policies aim to strengthen institutional capacity and promote collaboration among universities, research institutions, private-sector organizations, and government agencies. By encouraging interactions among these stakeholders, research funding facilitates knowledge creation, enhances innovation, and supports advanced skills development within the framework of the National Innovation System (Freeman, 1987; Lundvall, 1992; Organisation for Economic Co-operation Development, 2015). This study is theoretically grounded in the integration of National Innovation Systems (NIS) theory, Mission-Oriented Innovation Policy (MOIP), and Relational Governance to explain the strategic and collaborative mechanisms through which public research funding contributes to sustainable development outcomes. The National Innovation Systems perspective emphasizes the importance of interactions among universities, research institutions, industry, and government in fostering innovation, knowledge creation, and skills development (Freeman, 1987; Lundvall, 1992).

MOIP provides a framework for understanding how PMUs strategically direct research and innovation investments toward addressing societal challenges and achieving national development priorities, including digital transformation and the SDGs (Mazzucato, 2018). Rather than merely funding research projects, mission-oriented policies coordinate innovation activities across sectors to generate broad social and economic impacts.

The principles of Relational Governance complement these perspectives by emphasizing collaboration, trust, shared decision-making, and knowledge exchange among stakeholders involved in research and innovation programs. Such collaborative relationships enhance the translation of research outcomes into practical applications, strengthen institutional capacity, and support workforce development through the creation of new knowledge, skills, and innovation capabilities (Dyer and Singh, 1998; Poppo and Zenger, 2002). The conceptual framework for this study can be presented in Figure 1.

**Figure 1:**
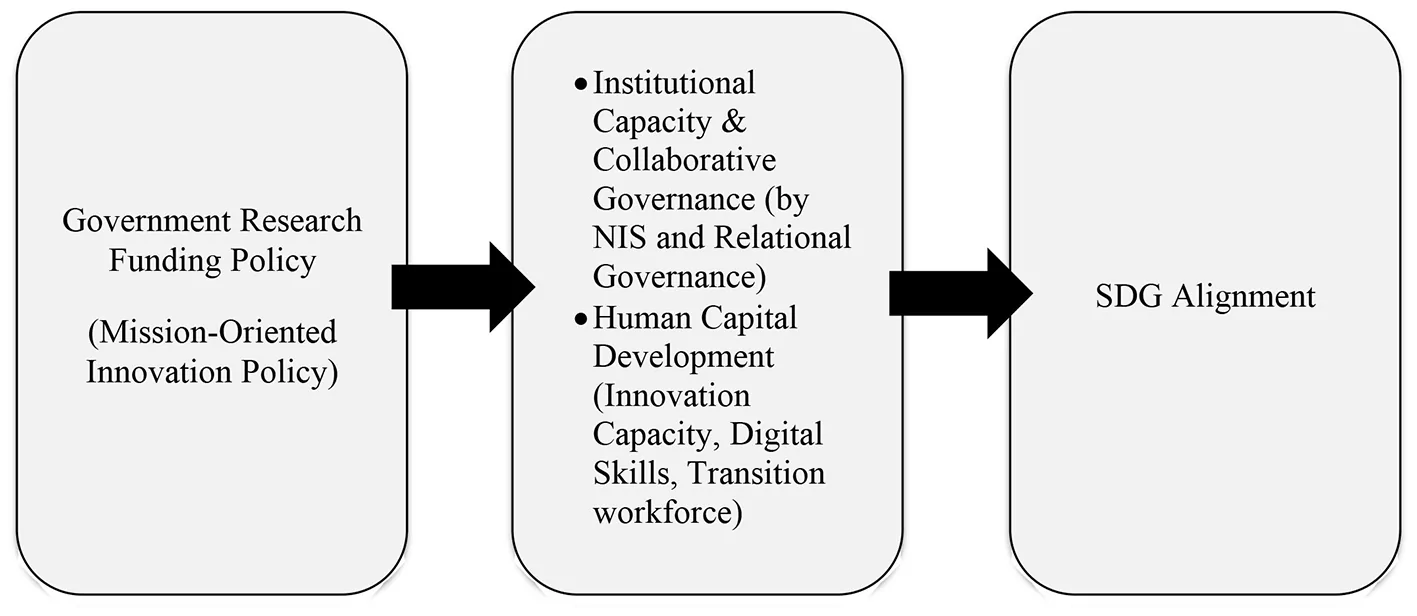
Conceptual framework.

Building on this integration, three propositions guide the empirical analysis that follows. Proposition 1: where PMUs operate as NIS intermediaries with an explicit mission-oriented mandate (MOIP), research funding is more likely to be directed toward workforce-relevant capability priorities than under funding models organized solely around disciplinary or academic-excellence criteria. Proposition 2: the extent to which PMU-mediated funding translates policy mandates into measurable capability outcomes depends on the strength of relational-governance mechanisms trust, iterative dialogue, and shared interpretation of goals between PMUs and the actors who implement funded activities. Proposition 3: indicators that require coordination across multiple agencies will show weaker achievement than indicators within a single PMU's direct operational control, because cross-agency coordination places greater demands on relational governance than intermediary-level coordination alone can supply. These propositions are examined against the empirical findings in the sections that follow.

The contribution of this article is therefore two-fold. Empirically, it offers a policy-system interpretation of Thailand's research-funding model as a vehicle for workforce-relevant capability development. Conceptually, it integrates National Innovation Systems theory, MOIP, and Relational Governance into a single analytical lens summarized in Figure 1 and uses that lens to explain how a research-funding intermediary can act as a driver of developmental alignment between innovation investment, human-capital formation, and SDG-oriented societal goals.

### 2.1 Core concepts: policy alignment, outcomes alignment, capability formation, and workforce transitions

Four concepts recur throughout this article and are defined here so that they can be applied consistently to the empirical findings. Policy alignment refers to the degree to which research-funding policies, governance structures, funding priorities, and implementation mechanisms are intentionally designed to support the objectives of the SDGs; it reflects the coherence between strategic policy goals and the institutional arrangements through which research resources are allocated and governed (Howlett, 2019; OECD, 2020). Outcomes alignment, in contrast, refers to the extent to which these governance arrangements generate observable results changes in research capacity, human-capital development, workforce competencies, interdisciplinary collaboration, research quality, innovation performance, and broader societal impacts that can reasonably be attributed to those governance arrangements (OECD, 2023; UNESCO, 2021). Section 4 below indicates, for each set of findings, whether the evidence speaks primarily to policy alignment or to outcomes alignment. Capability formation denotes the process by which individuals, organizations, and the wider research-funding system acquire and accumulate the skills, knowledge, and institutional capacities researcher competencies, organizational routines, and inter-organizational coordination capacity needed to sustain innovation-driven and future-workforce-oriented development over time. Workforce transitions refers to the structural shifts in labor-market demand and required competencies driven by digitalisation, demographic change, sectoral restructuring, and green and technological transformation that create the need for national HRD and research-funding systems to continuously reskill and redeploy human capital (OECD, 2019a; Nedelkoska and Quintini, 2018).

## 3 Methodology

This article draws on a completed national policy-monitoring study that examined the performance and implementation of key results indicators (“Key Results” or “KRs,” following the terminology used in the original TSRI monitoring framework) under Thailand's Science, Research and Innovation Plan (2020–2025), with a particular focus on Programmes P1–P5 and P 16**—**Progrmme1: “Create Systems to Produce and Develop Quality Human Resources,” Program 2: “Produce High-Quality Human Resources to Support the Eastern Economic Corridor (EEC),” Program 3: “Promote Lifelong Learning and Develop Future Skills,” Program 4: “Promote Intellectual Development as a Foundation for Thailand's Advancement Toward AI for All,” Program 5: “Promote Frontier Research and Basic Research in Areas Where Thailand Has Capacity,” and Program 16: “Reform the Higher Education, Science, Research, and Innovation System.” The original study employed an ex-post evaluation design conducted exclusively after the completion of the 2020–2022 implementation cycle. This enabled a retrospective analysis of target achievement, implementation dynamics, and systemic performance patterns across PMU-based research-funding systems.

The objective of each program is clearly specified. For example, the objective of Program 1 is to create factors that facilitate and promote the development of high-quality human resources, particularly among researchers, scientists, engineers, technology managers, foremen, and other highly skilled personnel. The program also aims to retain top talent, promote Work-Integrated Learning, and establish a system that accommodates master's and doctoral graduates. Additionally, it seeks to create mechanisms to attract Thai experts living overseas back to Thailand, as well as systems that encourage the exchange of human resources between domestic organizations. Within the research funding system, the objectives of each program are further defined through KRs, such as:

- **KR 1.1.1:** Develop a system that can be implemented for forecasting and planning HRD within the research system, aligned with the country's needs amid global change.
- **KR 1.1.2:** Develop a system for producing and supporting career pathways for researchers, ensuring continuity in research work, with the goal of increasing the number of researchers to 25 per 10,000 population.
- **KR 1.1.3:** Develop a joint or collaborative system between higher education institutions and private organizations to equip graduates and new graduates with skills that meet labor market demands, with at least 70% of graduates achieving this standard.
- **KR 1.1.4:** Establish a system to attract and support the exchange or rotation of researchers and experts between Thailand and foreign countries through position mobility, involving at least 1,000 personnel.

The original study employed an ex-post evaluation design, conducted upon the completion of the 2020–2022 implementation cycle, enabling retrospective analysis of target achievement, implementation dynamics, and systemic performance patterns across the PMU-based research-funding architecture.

The empirical foundation of the study rested on a mixed-methods evaluation design, integrating quantitative performance assessment with qualitative institutional inquiry integrated quantitative performance assessment with qualitative institutional inquiry. The quantitative analysis examined 376 program-level performance agreements, annual reports submitted to the PMUs, and TSRI-funded research reports. The qualitative component drew on field investigations of 30 research programs. A multistage sampling approach was employed: purposive sampling was used to invite five PMU administrators and ten staff for in-depth interviews. A combination of purposive and random sampling was used to recruit participants for the qualitative data collection. Focus group discussions were conducted with 10 experts based on qualification criteria specification. In addition, the interviews were conducted with 40 funded research project leaders who were selected according to predefined program eligibility criteria. A further focus group discussion was conducted with 30 research project leaders who had received research funding.

The quantitative component employed descriptive statistics including frequency distributions, percentage scores, and weighted mean calculations to evaluate programme-level target achievement, indicator alignment, and coherence within the PMU-based funding policy. Content validity for each key results indicator was established through the Index of Item-Objective Congruence (IOC), developed by Rovinelli and Hambleton (1977), calculated as *IOC* = Σ*R/N*, where *R* denotes expert scores (+1 for alignment, 0 for uncertainty, −1 for non-alignment) and *N* denotes the total number of experts. A minimum panel of three subject-matter experts evaluated each indicator against programme outputs. Indicators achieving an IOC score of 0.50 or above were classified as meeting the congruence threshold and proceeded to objective performance assessment; those below 0.50 were excluded from further analysis. Qualified indicators were then assessed against declared programme targets using a five-level coherence scoring rubric, ranging from 0 (not aligned) to 4 (fully aligned), with scores weighted equally across projects within each indicator to produce aggregated achievement percentages. Table 1 Presents an example of the scoring instrument used to assess the policy alignment of the KRs based on weighted criteria.

**Table 1:** Example of coherence scoring criteria.

Level	Description of the coherence criteria
4	Comprehensive information systems are in place and can be effectively used to forecast and plan workforce development needs for the research system and national demands in response to future global changes.
3	Meets the requirements of Levels 1–2, and pilot implementation of a workforce demand forecasting system for the research system and national needs under future global changes has been conducted.
2	Meets the requirements of Level 1, with integration and coordination among relevant agencies to drive the development of a workforce demand forecasting system for the research system and national needs under global changes.
1	Evidence exists of preliminary efforts to forecast workforce development needs for the research system and national demands under global changes.
0	Not aligned.

The qualitative component drew on four complementary sub-approaches: (1) focus-group discussions and expert opinion panels with PMU executives, programme leaders, funding recipients, and policy stakeholders; (2) in-depth interviews structured around internal organizational factors derived from the McKinsey 7S administrative management framework (Shaqrah, 2018; Alshaher, 2013) encompassing structure, strategy, systems, skills, style, staff, and shared values; (3) documentary analysis of programme reports, performance agreements, and official records submitted by PMUs to TSRI; and (4) participatory review sessions involving TSRI staff, enabling iterative knowledge exchange throughout the analytical process. The total numbers and the sampling strategies can be shown in Table 2. Qualitative material was analyzed thematically: interview and focus-group data were coded first deductively against the McKinsey 7S framework used to structure the interview protocol, and then inductively, to capture cross-cutting themes not anticipated by the framework (for example, the interpretive-broker role of PMUs as presented in the finding).

**Table 2:** Numbers of participants qualitative data collection.

Participants	Recruitment processes	Purposes	Number of participants
Experts	Qualification specification	Experts were purposively selected based on their qualifications to verify the validity of the research instruments and outcomes in order to:	10
		• Assess outcome alignment,	
		• Assess the validity of the rubric scoring for policy alignment, and	
		• Assess the rubric scoring for the McKinsey 7S dimensions.	
TSRIS, PMUs staff	Purposive sampling	Focus-group discussion and expert opinion interview	10
Recipient of research funding programs (Project leaders and team members.)	Purposive sampling: Research Programs with achieved outcome- alignment.	In-depth interview and focus-group discussion. (Field investigation).	12
Recipients of research funding	Purposive Sampling: Research Programs and Projects with High Level Outcome-alignment	Focus-group discussion and expert opinion panels (20 in Central region and the East, 20 in the North, and 20 in the Northeastern regions).	60
Recipients of research funding	Snowball Sampling: Experts Recommendation for Research Programs and Projects with Outstanding Outcomes	Online interview	40
Total			132

The principal documentary sources drawn upon in the original monitoring study and in the present re-analysis, and their role in the evaluation, are as follows. The primary source is the national monitoring report itself [Science, Research and Innovation Strategy and Policy Mission Group, Thailand Science Research and Innovation (TSRI), 2021], which records KR-level target statements, IOC expert-panel ratings, and rubric-based achievement scores (as summarized in Table 1) for each of Programmes P1–P5 and P16. This is supplemented by programme-level performance agreements and annual reporting documents submitted by PMUs to TSRI, and by the regulation establishing the PMU system (National Higher Education, Science, Research and Innovation Policy Council, 2019).

The analytical process followed an iterative five-stage sequence—data collection and cleansing, IOC-based congruence assessment, objective and subjective performance evaluation, gap identification, and synthesis incorporating two nested learning loops: a first-order loop examining the governance relationship between TSRI and the PMUs, and a second-order loop examining PMU interactions with grant recipients. This design is consistent with multi-level adaptive learning frameworks in which governance capacity emerges through iterative institutional feedback rather than linear programme delivery (Pahl-Wostl, 2009).

Building on this empirical foundation, the present article does not replicate the monitoring exercise, but instead re-interprets its findings through an analytic and conceptual lens. The study is positioned as a policy-learning case study, examining how indicator-driven research-funding governance influences national human-capital alignment in the context of future-workforce transitions and SDG-oriented development priorities. Methodologically, the re-analysis follows an interpretive policy-analysis approach, synthesizing three strands of evidence: (1) empirical patterns of programme outcomes and institutional drivers identified in the original study; (2) observed areas of alignment and misalignment between funding mechanisms and capability-development objectives; and (3) insights from international scholarship on human-capital systems, innovation-system governance, mission-oriented policy, and sustainable development. To connect the empirical findings and the analysis of policy alignment was conducted using scoring rubrics and the outcome. Evaluate the success of each project by comparing the achieved results with the target values specified in the performance agreement. The level of achievement was calculated and expressed as a percentage. Then combine the percentage success levels of all projects under the same KR and calculate the average to determine the overall percentage achievement of each Key Result's key performance indicator. The calculation of percentages and the averaging process consist of three steps: 1) Determine the weighted mean (weighted average) for each project, expressed as a percentage, by assigning equal weight to all projects under the same key performance indicator. 2) Assess the success of each project against the target values specified in the performance agreement and express the results as percentages. After that combine the percentage achievement of all projects to determine the overall percentage achievement of the key performance indicator. Calculation procedure is shown in Table 3.

**Table 3:** The calculation procedure for the outcome–alignment.

Symbol	Definition
*D*	Total number of projects implemented under a given Key Result (KR) in a given fiscal year (D ≥ 0)
*i*	Index of a project within the same KR and fiscal year, where i = 1, 2, …, D
*F_*i*_*	Percent completion (success rate) of project i (0 ≤ Fi ≤ 100)
*G_*i*_*	Percent contribution to the KR's success rate attributable to project i
*C*	Overall percent success rate of the KR for that fiscal year

**Calculation formulas**

1 Percent contribution to KR success from project i


$$\begin{array}{l} G_{i}=\left(100/\text{D}\right)\times \left(F_{i}/100\right) \end{array}$$ (1)

2 Overall percent success rate of the KR for the fiscal year

The overall percentage achievement of each Key Result (KR) was subsequently calculated as shown in Equation 2.


$$\begin{array}{l} C=\Sigma G_{i}=\left(1/D\right)\times \Sigma F_{i}. \end{array}$$ (2)

where the summation Σ runs from *i* = 1 to *D* (i.e., over all projects under the same KR and fiscal year).

**Condition:** If *D* = 0 (no projects implemented under the KR in that fiscal year), *C* is defined as 0 to avoid division by zero in Equation 1.

This study is explicitly bounded in scope: it does not constitute an organizational audit of individual PMUs, nor does it evaluate the internal operational processes or programme-level impact of any single funding programme. Rather, the key results indicators serve as the unit of analysis, with the analytical focus directed toward systemic governance factors institutional, coordination, and policy-design variables that mediate indicator performance across the PMU-based research-funding architecture. This methodological stance enables the article to move beyond performance description toward theorizing broader governance and policy implications relevant to other national contexts in which research funding, workforce transformation, and sustainable-development agendas increasingly intersect.

## 4 Results

The findings from the monitoring study reveal how Thailand's PMU-based research-funding framework has contributed to national human-capital development, while also exposing structural constraints that affect alignment with long-term workforce and SDG-oriented objectives. The results are presented in thematic areas.

### 4.1 Alignment between programme objectives and human-capital development priorities

Across Programmes P1–P5, the analysis demonstrates a strong structural alignment between programme objectives and Thailand's broader national priorities for human-capital development under the science, research and innovation policy framework. The monitoring report shows that the PMU system was explicitly designed to link research funding with capability formation, skills upgrading, and workforce responsiveness to global change, rather than treating research support as an isolated academic activity. The strategic orientation, policy coordination and policy implantation are the keys to manage the mission-oriented policy (Larrue, 2021). This intent is reflected in initiatives that strengthen the researcher pipeline, expand training ecosystems, and promote learning pathways that integrate higher education, research institutions, and industry sectors. For instance, the report highlights the role of MHESI as a coordinating intermediary matching private-sector skill requirements with education-sector provision, notably through the promotion of a Modular System that combines degree and non-degree learning options. This approach was introduced to ensure that workforce development remains flexible, responsive to emerging labor-market transitions, and aligned with long-term capability upgrading in strategic sectors of the economy (Martin and Godonoga, 2020; Ramsarup et al., 2023). In this sense, the PMU programmes did not simply produce graduates or project outputs, but were embedded in a broader national effort to steer human-capital development toward innovation-driven and future-workforce-oriented directions.

Quantitative performance evidence reinforces this overall pattern of alignment. The monitoring study reports that 100% of the assessed KRs achieved their target indicators across 376 funded projects, including major indicators such as KR 1.1.4, 1.3.5, 1.5.1, 1.5b.1, 1.5b.3, 1.5b.4, and indicators under Programme 16. KR codes follow the hierarchical numbering typically used in the original monitoring report, with the leading digit denoting the Programme and the subsequent digits denoting the specific Key Result and sub-indicator within that Programme's results framework. These KRs relate to areas such as research-talent development, capability strengthening within the research ecosystem, and alignment of programme outcomes with national workforce objectives. The overall performance across PMUs was assessed at 3.62 (out of a maximum of 4.00 on the five-level, 0–4 coherence rubric described in the Methodology, where scores of 3.00–3.99 are classified as “good level”), while the average conformity of indicators to programme goals was 3.57, suggesting that funded activities were generally implemented in accordance with strategic intentions and that the indicator framework succeeded in reinforcing programme–policy coherence. At the same time, the alignment score of 3.01 across five indicator levels reflects that coherence was present but uneven, indicating spaces where alignment was partial or dependent on programme interpretation rather than fully systemic. Taken together, these results illustrate that the PMU-based funding structure provided a meaningful institutional mechanism for translating national HRD priorities into research-linked activities and outputs (Howells, 2006; Smits and Kuhlmann, 2004).

Beyond indicator achievement, the findings also point to substantive contributions to longer-term capability trajectories within Thailand's human-capital system. The report identifies several emerging priority directions for future programme design, including: (i) development of up-to-date national databases of researchers and specialist expertise, to support workforce planning and knowledge-ecosystem mapping; (ii) creation of systems to attract and retain young researchers, in response to demographic and generational transitions within the research profession; and (iii) strengthening of industry–research partnerships and sector-aligned training ecosystems, particularly in fields linked to innovation-intensive production. These priorities reflect recognition within the PMU system that research funding contributes not only to knowledge production, but also to the cultivation of human-capital infrastructure necessary for national competitiveness and SDG-oriented development pathways. They also demonstrate that the programmes generated institutional learning about the types of investments and governance arrangements required to sustain future-oriented workforce capabilities.

However, despite this overall pattern of alignment, the analysis also reveals variation in the depth and quality of alignment across programmes and indicators. A number of indicators remained highly activity-based, focusing on counts of participants, projects, or training events, rather than on capability outcomes such as skill utilization in employment, research-career progression, or measurable contributions to workforce transformation. While such output indicators supported accountability and short-term performance reporting, they were less effective in capturing the developmental nature of human-capital formation, where impacts unfold over multiple years and depend on institutional, economic, and labor-market conditions. As a result, several programmes could demonstrate target achievement in quantitative terms, yet still faced difficulty evidencing how investments translated into sustained capability upgrading or structural workforce change. This tension reflects a broader systemic challenge in indicator-based governance frameworks, where the need for measurable delivery must be balanced against the inherently longitudinal character of workforce and skills development.

Furthermore, the indicators that required cross-programme or cross-agency integration—such as those linking education, labor, and innovation systems—showed weaker alignment with long-term capability outcomes. Fragmented responsibilities, differing interpretations of indicator intent, and uneven data-tracking capacity made it harder for some programmes to demonstrate coherent contributions to broader workforce transitions. In such cases, alignment existed at the level of policy rhetoric and programme intention, but was more limited at the level of system-wide coordination and cumulative capability impact. These findings suggest that while Thailand's PMU system has created a strong institutional foundation for aligning research funding with human-capital development, further refinement in indicator design, coordination mechanisms, and longitudinal tracking will be necessary to strengthen the connection between programme outputs, workforce transformation, and SDG-oriented development objectives.

### 4.2 Indicator performance and variation in achievement across programmes

The performance analysis revealed uneven achievement across key indicators, reflecting differences in programme design, institutional arrangements, and the nature of expected outcomes. Indicators linked to programme delivery, training participation, and research-talent engagement tended to perform strongly. These indicators were generally embedded in programmes with clear implementation mandates, established delivery models, and direct control by the responsible PMUs and project teams. As reported in the study, several programmes demonstrated high completion rates for training activities, scholarship schemes, and researcher-development initiatives, with performance scores in the “good” range and full achievement of key output-based targets across multiple indicators. This pattern suggests that the PMU system was particularly effective in supporting indicators that emphasized quantifiable participation outputs and short-cycle programme execution, where progress could be closely managed within organizational boundaries and monitored through established reporting mechanisms.

By contrast, indicators requiring cross-agency coordination, behavioral change among multiple stakeholders, or systemic ecosystem development were less likely to reach their intended performance levels. The report notes that indicators aimed at fostering integration across higher education, industry, labor-market institutions, and innovation actors often progressed more slowly or achieved only partial results. These indicators typically involved objectives such as aligning curricula with industry demand, coordinating talent mobility across sectors, or building multi-institutional workforce ecosystems outcomes that depend on shared interpretation of goals, complementary incentives, and sustained collaboration across organizational jurisdictions. In several cases, PMUs reported difficulties not in willingness to cooperate, but in the practical alignment of roles, expectations, and resources across participating agencies, which limited the speed and depth of progress achieved during the assessment period.

The performance variation was also influenced by differences in indicator measurability and data-system readiness. Indicators that relied on simple quantitative reporting such as counts of trainees, workshops, or scholarships benefitted from relatively mature monitoring systems and clear evidence frameworks. In contrast, indicators that sought to capture more complex system-level outcomes, such as capability utilization, institutional integration, or workforce-ecosystem change, faced challenges due to inconsistent definitions, limited longitudinal data, and fragmented information flows across agencies. The report highlights instances where implementing organizations interpreted indicator requirements differently or employed divergent performance-tracking methods, leading to variation in reported achievement and difficulty aggregating results at the national level. These challenges illustrate that even where commitment and effort were evident, institutional heterogeneity in monitoring capacity constrained the coherence of indicator-based steering.

The Thai experience therefore suggests that indicator frameworks are effective for driving delivery and accountability, but require complementary strategies such as relational coordination, shared learning platforms, and adaptive policy interpretation when the desired outcomes involve system-wide human-capital transformation rather than discrete programme outputs.

### 4.3 Factors supporting successful implementation within the PMU system

The qualitative evidence from interviews, review workshops, and programme documentation points to a set of institutional and governance characteristics within the PMU system that were particularly important in enabling successful implementation and the achievement of human-capital development objectives. A central factor was the flexible intermediary role of PMUs, which allowed programme managers PMU-employed staff responsible for the day-to-day strategic oversight of a programme's funded projects, distinct from the project leaders who had received research funding,to balance national policy direction with the practical realities of implementation across diverse institutional and regional contexts. Rather than acting as purely administrative funding agencies, the PMUs functioned as strategic coordinators and translators of policy intent, supporting project teams in interpreting objectives, tailoring activities to local needs, and responding to unforeseen implementation challenges. This intermediary positioning enabled programmes to move beyond rigid compliance-based implementation and instead adopt context-sensitive delivery approaches, particularly in initiatives related to workforce development, training ecosystem expansion, and researcher-capacity strengthening.

A second enabling factor was the presence of a mission-oriented funding logic, which provided a shared strategic frame across projects and stakeholders. Programmes organized around clearly articulated national priority themes such as talent development for innovation sectors, upgrading workforce capabilities in emerging industries, or strengthening research-to-industry linkages created a sense of collective direction and policy coherence, even when specific activities varied across implementing organizations. This mission orientation helped align academic institutions, research bodies, and partner organizations around common capability-development goals, reducing fragmentation and encouraging collaboration in areas where previously institutional incentives may have been weak or uncoordinated. It also contributed to a stronger focus on problem-solving and outcome relevance, rather than treating projects as isolated or output-driven exercises.

The study further highlights the role of programme managers and specialist expert networks in mediating between policy expectations and implementation realities which are appointed by PMUs. When respondents were asked about the key success factors for PMU management and the guidelines for achieving their work units' OKRs, the responses were coded and classified using the McKinsey 7S framework. The findings revealed that PMUs A and B (newly established organizations) scored highly in areas related to strategy and work style. Specifically, their strengths included close internal collaboration, teamwork across the organization, and the establishment of a consortium allowing external experts to contribute to funding policy missions. In contrast, PMUs C and D (bureaucratic organizations) scored highly in teamwork, external expert consortia, and a working style rooted in the benefits of their legacy organization. Consequently, strong networking emerged as a key strength for these two organizations.

Many of the more successful programmes were supported by managers and advisers with substantial professional experience in research management, workforce development, or sector-specific ecosystems. These actors were able to interpret objectives in a developmental rather than procedural manner, guide project leaders in refining implementation strategies, and facilitate access to relevant expertise and collaboration partners. Their role extended beyond monitoring and reporting; they acted as brokers of knowledge, relationships, and strategic learning, enabling projects to adapt to emerging needs while remaining aligned with programme purposes.

PMUs that actively cultivated iterative dialogue and relational engagement with project leaders, partner organizations, and beneficiaries demonstrated a stronger capacity to translate funding objectives into meaningful human-capital outcomes. Regular consultations, reflective learning activities, and ongoing feedback loops allowed programme teams to surface implementation constraints, explore alternative approaches, and adjust activities in ways that strengthened impact. These relational governance mechanisms complemented rather than replaced the formal indicator and reporting structures. While indicators provided accountability and direction, it was the quality of interaction, trust-building, and shared interpretation of goals that supported deeper forms of capability development and workforce transformation within the funded initiatives.

### 4.4 System-level constraints and gaps affecting long-term capability outcomes

Despite the positive contributions identified in earlier sections, the findings also reveal several structural constraints within the wider governance and implementation environment that limit the extent to which research-funded programmes can generate sustained capability and workforce-system transformation. A key challenge relates to fragmentation across programmes, agencies, and funding mandates, which reduced opportunities for horizontal integration of human-capital initiatives. Although many programmes pursued similar objectives such as researcher development, skills upgrading, or innovation-ecosystem strengthening coordination mechanisms were often limited to project-level interactions rather than system-level alignment. As a result, some workforce initiatives operated in parallel rather than in synergy, creating risks of duplication, uneven coverage, and weak cumulative impact across regions and sectors. This fragmentation also made it more difficult to build coherent capability pathways linking education, research, and labor-market outcomes over time.

A second structural constraint concerns the design and orientation of performance indicators, which tended to privilege short-term, measurable outputs such as activity counts, participation volumes, or programme completion rates over indicators capturing developmental trajectories and capability maturation. While such indicators are valuable for accountability and monitoring, they do not adequately reflect processes such as skill utilization in employment, research-career consolidation, institutional learning, or changes in organizational capability cultures. Consequently, programme achievements were often visible in output terms, but less visible in relation to how investments contributed to deeper and longer-term workforce transformation. This imbalance reflects a broader difficulty in translating complex capability-building processes into performance metrics that are both meaningful and administratively feasible.

A third gap relates to the absence of systematic post-programme tracking and longitudinal follow-up mechanisms. Once projects or training cycles were completed, data on participants, institutions, and capability outcomes were typically dispersed across organizations or not collected beyond the reporting period. This limited the ability of policymakers to assess whether research-funded human-capital initiatives generated lasting effects on employment trajectories, innovation performance, institutional practices, or SDG-related societal outcomes. Without longitudinal evidence, opportunities for double-loop learning such as revising programme assumptions, redesigning funding instruments, or refining strategic priorities remained constrained, even where stakeholders recognized the importance of long-term impact assessment.

## 5 Discussion

The findings from the monitoring study point to a set of broader insights about how national research-funding systems shape human-capital development trajectories in the context of future workforce transitions and sustainable-development objectives. Research funding governance has increasingly been recognized as a strategic policy instrument for advancing the SDGs by directing research investment, shaping institutional priorities, and strengthening national research capacity (United Nations, 2015; UNESCO, 2021). While growing attention has been given to aligning research funding with the SDGs, the literature often conflates policy intentions with the outcomes that these policies ultimately produce. To enhance conceptual precision, this study distinguishes between policy alignment and outcomes alignment, recognizing them as related but analytically distinct dimensions of research funding governance. In line with emerging international scholarship on mission-oriented research governance and skills-ecosystem transformation, the Thai case illustrates that research funding is not only an instrument for knowledge production, but also a strategic lever for capability formation, labor-market adaptation, and institutional learning (Vandeweyer et al., 2020; Gennrich, 2017). However, the results also show that the extent to which these contributions materialize depends on how governance structures, indicators, and coordination mechanisms mediate the relationship between programme activities and long-term workforce outcomes (Mazzucato, 2018; Borrás and Edquist, 2013).

### 5.1 Research-funding governance as a driver of capability formation

The alignment observed between programme mandates and human-capital development priorities reflects the theoretical proposition that research-funding systems operate not merely as financial allocation instruments, but as core institutional components of the National Innovation System (NIS). Within the NIS perspective, capability formation and innovation performance emerge from interactive relationships among state agencies, research organizations, firms, and labor-market institutions, rather than from technological investment alone (Lundvall, 2016, 1992; Nelson, 2013). In this sense, Thailand's PMU-based structure exemplifies the function of research-funding intermediaries as system coordinators, shaping incentive structures, guiding organizational behavior, and steering resources toward priority capability domains such as researcher development, sector-specific reskilling, and innovation-ecosystem strengthening. This interpretation is consistent with the view that capability upgrading is co-produced through innovation-system governance, where funding logics, programme architectures, and institutional interpretations collectively influence the trajectory and depth of workforce transformation, rather than being determined solely by education or labor-market policy instruments (Lundvall, 2010; Wieczorek and Hekkert, 2012).

The Thai case also resonates with insights from MOIP, which argues that funding programmes organized around societal or strategic missions can reorient public investment toward long-term transformational objectives (Kirchherr et al., 2023; Mazzucato, 2018; Foray, 2018). The PMU framework reflects a mission-oriented design in which research funding is deliberately linked to the development of national strategic capabilities—including digital competencies, research-talent pipelines, and skills for innovation-intensive sectors. From a theoretical standpoint, missions function as direction-setting devices, aligning heterogeneous actors around shared developmental priorities while creating protected spaces for experimentation, learning, and capability accumulation (Weber and Rohracher, 2012). This suggests that research-funding governance does not simply enable project execution; rather, it actively structures patterns of capability formation and workforce specialization in alignment with broader national development strategies (Schot and Steinmueller, 2018; Edler and Fagerberg, 2017).

At the same time, the observed variation in indicator performance reinforces arguments from skills-ecosystem and capability-formation literature, which emphasize that workforce capabilities emerge from relational, context-embedded processes, rather than from linear training or investment inputs (Ramsarup et al., 2023; Finegold, 1999). Indicators associated with participation volumes and activity delivery were more readily achieved because they operated within bounded organizational environments, whereas indicators involving collaboration, cross-institutional integration, and systemic change depended on interactive learning, trust-building, and negotiated interpretation of objectives. These dynamics echo institutionalist perspectives on governance, which argue that policy outcomes are shaped not only by formal rules and instruments, but also by interpretive practices, professional norms, and coordination relationships among implementing actors (March and Olsen, 2006).

From this perspective, indicators and performance-management tools alone are insufficient to generate capability transformation. Capability outcomes improve when programmes combine clear strategic intent with adaptive and reflexive governance practices, enabling actors to reinterpret goals, share knowledge, and adjust implementation strategies in response to emerging challenges. The empirical evidence from Thailand illustrates how programme managers, expert networks, and PMU intermediaries functioned as “interpretive brokers,” mediating between policy expectations and implementation realities—a dynamic consistent with governance theories that stress the importance of collaborative interpretation and deliberative coordination in complex policy systems (Ansell and Gash, 2008). In this configuration, research-funding governance becomes not only an administrative instrument, but a strategic arena through which capability formation is shaped, negotiated, and sustained (Hajer and Wagenaar, 2003).

### 5.2 Critical reflections on indicator-driven governance and measurement

A further point of interpretation concerns the finding, reported above, that 100% of assessed KRs achieved their target indicators. Taken at face value, this figure could be read as evidence of uniformly strong programme performance; however, consistent with the broader literature on governance-by-indicators, such results warrant critical interpretation rather than straightforward acceptance (Bevan and Hood, 2006; Dahler-Larsen, 2014; Merry, 2011). At least three interpretive caveats apply. First, because indicators falling below the 0.50 IOC congruence threshold were excluded from further analysis before performance scoring, the reported achievement rate describes only those indicators judged sufficiently valid and well-specified to proceed to assessment, not the full universe of originally proposed indicators; this selection step may itself inflate apparent success rates. Second, full target achievement is more readily attainable for indicators that are activity-based and within the direct operational control of a single PMU (e.g., counts of trainees or scholarships) than for indicators requiring cross-agency coordination precisely the pattern observed in the variation in indicator performance reported above which suggests that the aggregate 100% figure may mask a “ceiling effect,” in which targets for the most tractable indicators were met while more demanding, systemic indicators were disproportionately excluded, under-specified, or set conservatively. Third, as in many public-sector performance regimes, administrative and reputational incentives to report full compliance cannot be ruled out as contributing to observed achievement rates, independent of underlying capability change (Bevan and Hood, 2006). None of these caveats imply that the reported achievements are inaccurate; rather, they indicate that indicator-based performance reporting is, on its own, an imperfect proxy for the deeper, longer-term capability formation that this article seeks to examine reinforcing the case, developed in the next sub-section, for dual-layer performance architectures that combine output accountability with developmental, longitudinal evaluation.

### 5.3 The challenge of linking short-term outputs to long-term workforce transitions

The results highlight a structural governance tension widely recognized in evaluation and public-management theory: the misalignment between short-term, output-oriented accountability mechanisms and the longitudinal, cumulative nature of capability development. Indicators centered on participation counts, training delivery, and project completion align closely with what results-based management frameworks identify as outputs and short-term outcomes, which are highly measurable and closely tied to programme activities. By contrast, indicators associated with ecosystem integration, institutional learning, behavioral change, and labor-market transformation correspond to intermediate or developmental outcomes, which evolve through iterative, context-embedded processes and are therefore less immediately observable or attributable to single interventions (Manna et al., 2018; Kusek and Rist, 2004).

This finding is consistent with critiques of governance-by-indicators, which argue that performance systems tend to privilege what is quantifiable and time-bounded over what is structural, relational, and path-dependent (Merry, 2011; Dahler-Larsen, 2014). From a capability-formation and skills-ecosystem perspective, workforce transformation depends not only on discrete training inputs but on capability utilization, organizational routines, career-path consolidation, and institutional adaptation—processes that align more closely with evolutionary and institutional theories of change than with linear programme-delivery logics (Ramsarup et al., 2023; Lundvall, 2016). The uneven performance of integration-oriented indicators observed in the study reflects this theoretical insight: outcomes requiring collective coordination, shared meaning, and cross-actor alignment cannot be generated or evidenced through target compliance alone, because they depend on relational dynamics and cumulative institutional adjustment.

From a policy-learning and developmental-evaluation standpoint, these findings suggest that research-funding governance requires dual-layer performance architectures. One layer ensures operational accountability and delivery discipline, while a second layer supports reflexive, developmental, and longitudinal evaluation capable of tracing capability trajectories, cross-programme interaction effects, and emergent institutional capacities over time (Argyris and Schön, 1996). Such arrangements align with theories of double-loop learning and adaptive governance, which emphasize the importance of revisiting assumptions, reframing objectives, and feeding experiential learning back into programme and system redesign (Pahl-Wostl, 2009).

Without these complementary evaluative layers, performance regimes risk reinforcing compliance-centered behaviors, incentivising organizations to prioritize indicator fulfillment over capability deepening and ecosystem strengthening. In such circumstances, investments may generate strong activity records while contributing only partially to future-workforce resilience, innovation-system transformation, and SDG-aligned societal outcomes (Bevan and Hood, 2006). The Thai case thus illustrates a broader theoretical challenge facing many national systems: the need to move from viewing performance as a short-cycle measurement exercise toward understanding it as a continuous, learning-oriented governance process grounded in the temporal and systemic nature of capability development.

### 5.4 Implications for SDG-aligned human-capital and workforce policy

Interpreted through the lens of the SDGs, particularly SDG 4 (Quality Education), SDG 8 (Decent Work and Economic Growth), SDG 9 (Industry, Innovation and Infrastructure), and SDG 10 (Reduced Inequalities) (United Nations, 2015), the Thai case illustrates both the transformative potential and structural limitations of aligning research-funding governance with human-capital and workforce-development objectives. From a theoretical standpoint, the SDGs function not merely as a set of global targets, but as a normative and coordination framework that encourages governments to integrate social, economic, and innovation-related priorities within a single policy architecture (Sachs, 2015; Fukuda-Parr and McNeill, 2019). Within this perspective, Thailand's PMU-based funding system contributes positively to SDG alignment by expanding learning pathways, strengthening research-talent ecosystems, and directing capability investments toward priority sectors and emerging knowledge domains. These dynamics are consistent with capability-based and inclusive-development approaches, which view human-capital development as a foundation for widening opportunity structures and enhancing societal resilience (Sen, 2014; Barreno-Alcalde et al., 2024).

However, the study also demonstrates that the realization of SDG-aligned benefits is constrained by fragmentation across policy domains, limited cross-sectoral integration, and weak post-programme tracking of social and labor-market outcomes. These challenges resonate with the literature on policy coherence for sustainable development (PCSD), which argues that achieving SDG-related transformations requires institutions capable of reconciling sectoral mandates, overlapping accountability structures, and competing policy logics (OECD, 2019b). In the Thai case, investments in research-driven capability building generated measurable programme outputs, yet the absence of systematic longitudinal evidence made it difficult to assess how these initiatives contributed to inclusive employment trajectories, equitable skill utilization, or reductions in capability gaps across regions and social groups (Vandeweyer et al., 2020). This reflects a broader tension in SDG governance between programme-level activity delivery and the macro-level structural transformations that the SDGs seek to advance.

From a multi-level governance perspective, the findings suggest that aligning HRD, research funding, and the SDGs requires more than symbolic alignment or output mapping. Instead, it demands institutional integration across education, research, labor, industry, and regional-development systems, supported by governance mechanisms capable of connecting micro-level programme achievements with meso- and macro-level workforce transitions (Bache and Flinders, 2004; Jordan and Lenschow, 2010). Such integration involves not only formal coordination structures, but also shared interpretive frameworks, cross-organizational learning processes, and evaluation architectures that trace distributional and inclusion effects of capability investments over time. The Thai experience indicates that this form of alignment is achievable but contingent—it depends on sustained coordination, policy coherence, iterative learning, and institutional commitment to SDG-oriented transformation, rather than on isolated programme success alone.

### 5.5 Transferable lessons for countries in similar contexts

Policy alignment concerns the design of governance systems funding criteria, evaluation frameworks, incentive structures, and capacity-building initiatives that explicitly target SDG-related objectives, while outcomes alignment concerns whether those governance arrangements demonstrably produce the intended results. Applying that distinction here, the Thai case indicates that research-funding governance arrangements generate observable results contributing to SDG-related objectives only when funding allocation processes, governance practices, and institutional support systems are intentionally designed to support those objectives and are matched by adequate implementation capacity. Evidence of improvements in researchers' competencies, workforce capability, or institutional research capacity is therefore interpreted here as evidence of outcomes alignment only when it can reasonably be attributed to governance arrangements that were intentionally designed for SDG-related objectives, rather than to factors external to the funding system.

Although empirically grounded in the Thai context, the insights generated from this study offer broader relevance for middle-income and transition-economy settings seeking to align research-funding governance with future-workforce and sustainable-development agendas. These lessons are most directly transferable to countries that share three structural features with the Thai case: (i) a middle-income, transition economy still building a diversified research base rather than an advanced, high-capacity innovation system; (ii) a centralized or semi-centralized state apparatus capable of establishing and resourcing intermediary funding agencies with real operational discretion; and (iii) an explicit policy mandate linking research investment to workforce and human-capital objectives, rather than research funding organized solely around disciplinary or academic-excellence criteria. Where these conditions do not hold, for example in highly decentralized federal systems, in economies with mature and diversified public research-funding landscapes, or where research funding is governed independently of workforce policy, the applicability of the specific institutional lessons identified here is likely to be more limited, even if the underlying analytical logic that mission clarity and intermediary capacity jointly shape capability outcomes remains relevant. From a comparative policy perspective, such contexts typically operate within institutionally fragmented innovation systems, characterized by uneven capability bases, transitional labor markets, and evolving state capacities (Bugge et al., 2024; Evans, 2014). The findings of this case study indicate that efforts to strengthen the relationship between research funding and human-capital development are most effective when national systems incorporate three mutually reinforcing design elements that are also supported in the literature.

First, mission-oriented funding structures play a critical role in embedding workforce and capability goals within innovation policy. Consistent with mission-oriented innovation and developmental-state traditions, such structures help articulate strategic directionality, align heterogeneous actors around common societal priorities, and channel public investment toward capability accumulation in strategically significant domains (Mazzucato, 2018; Amsden, 2001). For countries facing technological disruption and industrial restructuring, mission-oriented approaches provide a governance mechanism for linking innovation policy, labor-market transformation, and skills formation in a coherent policy frame, rather than treating them as separate domains.

Second, the Thai case highlights the importance of intermediary institutions such as PMUs that are capable of relational coordination, adaptive problem-solving, and interpretive governance. Theoretical work on intermediation and collaborative governance suggests that such institutions serve as bridges across organizational boundaries, facilitating shared understanding, trust-building, and negotiated alignment among actors with diverse mandates and rationalities (Howells, 2006; Ansell and Gash, 2008). In contexts where bureaucratic fragmentation and capability asymmetries are common, intermediaries help convert strategic missions into implementable, context-sensitive practices, thereby increasing the likelihood that research investments translate into meaningful capability outcomes.

Third, the case underscores the value of performance frameworks that balance output accountability with evidence of long-term capability trajectories. This aligns with insights from developmental evaluation and policy-learning theory, which emphasize that transformation-oriented systems require evaluative architectures capable of supporting both delivery discipline and reflexive, longitudinal learning (Wu et al., 2017; Argyris and Schön, 1996). For countries in similar contexts, adopting such dual-layer performance systems can help avoid the risks of indicator-driven compliance behavior while strengthening the ability of policymakers to track how research-funding interventions contribute to workforce resilience, innovation-system upgrading, and inclusive development outcomes over time (Melin et al., 2015).

## 6 Conclusion

This article has examined how national research-funding governance can shape human-capital development and workforce transformation, using evidence from Thailand's PMU-based research-funding system under the national Science, Research and Innovation Plan. The analysis demonstrates that research funding functions not only as a mechanism for resource allocation, but as a strategic governance instrument within the National Innovation System, influencing patterns of capability formation, institutional coordination, and labor-market adaptation. Through the lenses of MOIP, skills-ecosystem theory, and developmental evaluation, the study shows that capability upgrading is co-produced through the interaction of funding logics, programme architectures, intermediary coordination, and relational governance practices, rather than through training investments or output-based performance mechanisms alone.

The findings highlight a dual reality. On the one hand, Thailand's mission-oriented, PMU-intermediated funding model has strengthened researcher pipelines, expanded learning pathways, and aligned capability investments with national development priorities. On the other hand, fragmentation, indicator-driven accountability, and limited longitudinal tracking continue to constrain the visibility of long-term workforce outcomes and SDG-related societal contributions. These tensions reflect a broader governance dilemma facing countries seeking to link research funding with future-workforce transitions: how to reconcile the administrative demands of measurable delivery with the temporal, relational, and systemic nature of capability development.

Based on this case, the article advances three broader policy-learning insights with relevance for other middle-income and transition-economy contexts. First, mission-oriented funding structures help embed capability and workforce priorities within innovation policy, providing strategic directionality in contexts of technological and economic uncertainty. Second, intermediary institutions capable of relational coordination and interpretive governance play a critical role in translating strategic missions into context-sensitive implementation and meaningful capability outcomes. Third, dual-layer performance architectures combining output accountability with developmental and longitudinal evaluation are essential for understanding how research-funding interventions contribute to cumulative capability trajectories and inclusive workforce transformation over time.

The contribution of this article is therefore two-fold. Empirically, it offers a policy-system interpretation of Thailand's research-funding model as a vehicle for workforce-relevant capability development. Conceptually, it brings together insights from innovation-system governance, mission-oriented policy, capability and skills-ecosystem theory, and policy-learning frameworks to argue that research-funding systems can act as drivers of developmental alignment between innovation investment, human-capital formation, and SDG-oriented societal goals.

Future research could build on this analysis by undertaking comparative case studies across countries, developing longitudinal datasets on capability trajectories, and exploring how political economy dynamics, institutional incentives, and actor coalitions shape reform pathways in research-funding governance. Such work would deepen understanding of how national systems can better harness research investment not only for technological progress, but for sustainable, inclusive, and future-ready workforce development.

Accordingly, this study argues that effective research funding governance represents a critical intermediary between strategic policy commitment and sustainable development outcomes. Policy alignment establishes the strategic direction and institutional conditions necessary for SDG-oriented research, whereas outcomes alignment provides empirical evidence of whether these governance arrangements successfully enhance human capital development and strengthen the research workforce. Analytically separating these constructs enables a more robust evaluation of governance effectiveness and contributes to a clearer theoretical understanding of the role of research funding systems in advancing the SDGs.

## Data Availability

The raw data supporting the conclusions of this article will be made available by the authors, without undue reservation.
