# Supplementary material for: Aligning national human resource development with future workforce transitions and the SDGs: policy lessons from Thailand's research-funding system
Source: Front Res Metr Anal. 2026 Sep 21;11:1830772. doi: 10.3389/frma.2026.1830772 (PMC13634869; doi:10.3389/frma.2026.1830772)
Supplement: Supplementary file 1 [file Table_1.docx]

**Appendix**

**Example of Data Analysis Outcomes for the Evaluation of Key Result Indicators**

**Table 1 Results of the Evaluation of Key Result Indicator (KR 1.1.2) under P I.**

| Year | Description of Key Result Indicator | Responsible Agency | Level of Alignment | % | Project Code / Achievement (%) |
| --- | --- | --- | --- | --- | --- |
| 2020 (B.E. 2563) | KRI 1.1.2: Establish a system for developing and supporting researchers' career pathways and continuous research career advancement, with the goal of increasing the number of researchers and developers to 25 per 10,000 population (1 system). | MHESI | 3 | 76.27 | 3–6/100 |
|  |  |  | 3 |  | 2/83.33 |
|  |  |  | 3 |  | 7/50 |
|  |  |  | 0 |  | 1/0 |
| 2021 (B.E. 2564) | KRI 1.1.2: Establish a system for developing and supporting researchers' career pathways and continuous research career advancement, with the goal of increasing the number of researchers and developers to 25 per 10,000 population (1 system). | MHESI | 3 | 66.00 | 132,134/100 |
|  |  |  | 3 |  | 131/80 |
|  |  |  | 3 |  | 135/50 |
|  |  |  | 0 |  | 133/0 |
| 2022 (B.E. 2565) | KRI 1.1.2: Conduct a system assessment and identify the needs of the national science, research, and innovation (STI) system that have implications for the country's competitiveness and development (1 system). | – | – | – | – |

**Note:** "-/-" indicates that no responsible agency was assigned to implement the program.
